# Recruitment of UvrBC complexes to UV-induced damage in the absence of UvrA increases cell survival

**DOI:** 10.1093/nar/gkx1244

**Published:** 2017-12-12

**Authors:** Luke Springall, Craig D Hughes, Michelle Simons, Stavros Azinas, Bennett Van Houten, Neil M Kad

**Affiliations:** 1 School of Biological Sciences, University of Kent, Canterbury CT2 7NH, UK; 2 Department of Veterinary Medicine, University of Cambridge, Cambridge CB3 0ES, UK; 3 School of Biological Sciences, University of Essex, Colchester CO4 3SQ, UK; 4 UPMC Hillman Cancer Center, Pittsburgh, PA 15213, USA

## Abstract

Nucleotide excision repair (NER) is the primary mechanism for removal of ultraviolet light (UV)-induced DNA photoproducts and is mechanistically conserved across all kingdoms of life. Bacterial NER involves damage recognition by UvrA_2_ and UvrB, followed by UvrC-mediated incision either side of the lesion. Here, using a combination of *in vitro* and *in vivo* single-molecule studies we show that a UvrBC complex is capable of lesion identification in the absence of UvrA. Single-molecule analysis of eGFP-labelled UvrB and UvrC in living cells showed that UV damage caused these proteins to switch from cytoplasmic diffusion to stable complexes on DNA. Surprisingly, ectopic expression of UvrC in a *uvrA* deleted strain increased UV survival. These data provide evidence for a previously unrealized mechanism of survival that can occur through direct lesion recognition by a UvrBC complex.

## INTRODUCTION

Genomes are constantly assaulted by both endogenous and exogenous agents resulting in an array of DNA lesions (1). Efficient DNA repair is therefore essential for survival of all organisms. Solar ultraviolet light (UV) can induce cytotoxic cyclopyrimidine dimers and 6–4 photoproducts (2,3). Removal of these UV-induced DNA lesions occurs through nucleotide excision repair (NER). This pathway is highly mechanistically conserved from bacteria to mammals and proceeds through the following discrete steps: damage recognition, damage verification, lesion processing and removal, repair synthesis and ligation. In bacteria, lesion recognition is achieved by UvrA and UvrB working in concert (4–12). UvrB verifies that the lesion is suitable for repair (13) and subsequently UvrA is ejected (6). The next stage in repair is incision; UvrC is recruited to the UvrB-bound lesion site and cuts the DNA backbone both 5′ and 3′ to the lesion on the same strand (14). The resulting 12–13 nt patch is removed by UvrD (DNA helicase II), which also recycles UvrC before the final stage of repair: DNA synthesis and sealing of the repair patch (15,16). In prokaryotes, DNA polymerase I uses the single stranded patch as a template for resynthesizing undamaged DNA which is subsequently resealed by DNA ligase (1,17).

Many questions remain about this mode of repair: including the physical search mechanisms employed, the method of partner recruitment and the repair intermediates formed (18). We have begun to address how lesions are found using single-molecule techniques. By fluorescently labelling single molecules of UvrA and UvrB, we were able to show that these proteins use a combination of three-dimensional (3D) jumping and one-dimensional (1D) sliding to scan the genome for lesions (11). These studies were performed on elevated micro-platforms-termed DNA tightropes, and enabled us to also study which complexes formed during repair. Our observations suggested, in agreement with bulk studies (9,13), that UvrA and UvrB formed a UvrA_2_B_2_ complex. In addition, UvrB and UvrC were also observed to form a complex independently of UvrA (19,20). This intriguing complex has been previously identified during bulk studies (21,22), but has shown no apparent repair efficiency on dsDNA *in vitro* (20,23,24). Therefore, the role of this complex has remained uncertain.

Here, using a combination of single-molecule imaging techniques *in vitro* and in living *Escherichia coli* cells, coupled with UV cell survival experiments, we investigate the occurrence and role of specific bacterial complexes in repair. We reveal evidence for the existence of a ‘repairosome’ complex (25,26) comprised of UvrA, UvrB and UvrC. Such a complex could greatly enhance the efficiency of repair by bringing all of the repair components together at the sites of damage. Using defined lesions on DNA tightropes, we were also able to define which complexes associate with damage. Surprisingly, we found that the complex of UvrB and UvrC (UvrBC) could locate lesions. *In vivo* fluorescence imaging of *E. coli* UvrA-null cells showed that eGFP-tagged UvrC binds to the genome when exposed to UV. In parallel, we also show that slightly elevated UvrC levels enhance the resistance of UvrA-null cells to low doses of UV. Together, these data suggest a mechanism of repair exists in cells suffering low levels of damage, in which UvrBC complexes have the capacity to locate genomic lesions independently of UvrA.

## MATERIALS AND METHODS

### Proteins and cell lines

All *in vitro* experiments were performed with *Bacillus caldotenax* Uvr proteins prepared as described in the Supplementary Data. Cell survival assays and live cell imaging were performed with *E. coli* (K-12 strain BW25113) KEIO cells and C-terminally eGFP tagged *E. coli* Uvr proteins were obtained as ASKA clones from the National Bioresource Project (NIG, Japan) (27–29). Details of expression levels are provided in the Supplementary Data.

### Cell survival assay

Lysogeny broth (LB) containing the appropriate antibiotic was inoculated from a 15% glycerol cell stock and grown overnight at 37°C; subsequently this was diluted into fresh LB and grown to OD_600_ 0.6. Aliquots of undiluted and three serial ten-fold dilutions of cells were either spotted or spread on LB-agar plates. Plates were then exposed to the stated doses of 254 nm UV to induce DNA damage and incubated overnight. To generate a UvrA-null control cell line (UvrA^−^) that contained an equivalent protein load and antibiotic resistance to those with Uvr proteins, we transformed UvrA KEIO cells with a plasmid containing the protein Yihf-eGFP, a protein unrelated to NER.

### Constructing damaged and single-stranded DNA tightropes

Incorporation of damage into the 16 μm long λ-DNA was achieved by using the nickase Nt.BstNBI strategy established previously (30,31). Details are provided in the Supplementary Data.

### Fluorescence imaging

#### Standard conditions

Unless otherwise stated all *in vitro* single-molecule experimental procedures were performed at room temperature in ABC buffer (50 mM Tris–HCl (pH 7.5), 50 mM KCl, 1 mM adenosine triphosphate (ATP) and 10 mM MgCl_2_, 10 mM Dithiotheitol (DTT)).

#### Single-molecule fluorescence imaging

For fluorescence imaging we constructed a microscope capable of imaging up to three colours simultaneously using a TripleSplit (Cairn Research, UK) optimized for 565, 605 or 655 nm quantum dots (Qdots). We used established protein labelling strategies which we previously demonstrated have no effect on protein activity (11,19,32) with a 4:1 excess of Qdot to protein to ensure Qdots were singly labelled (32). The same protein was not always labelled with the same colour Qdot to ensure that this did not introduce any bias. For more details of single-molecule imaging procedures and a description of the microscope used please refer to the ‘Supplementary Data’ section.

#### Live-cell imaging

LB was inoculated with cells from a 15% glycerol stock and grown overnight at 37°C, then diluted into fresh LB and grown to OD_600_ 0.6. One millilitre of cells were centrifuged, resuspended in fresh LB, diluted 1/20 in LB before 5 μl was deposited on 3% agarose/1× LB pads. Non-damaged cells were imaged immediately after immobilization. Damaged cells were exposed to 5 or 25 J/m^2^ UV (254 nm) and incubated at 37°C for 30 min to allow for an adequate SOS response (33,34) prior to imaging. Images were acquired at 10 frames per second (fps) for 10 s using the microscope described in the Supplementary Data.

### Data analysis

#### Quantum dot colocalization

Individual isotropic fluorophores emitting as a point source generate a fluorescent point spread function (PSF) orders of magnitude larger than the size of the source. When in focus this PSF approximates well to a Gaussian distribution with a width approximately the wavelength of light (35). Although the size of the Qdot is on the nanometer scale, the fluorescence it emits appears on the micron scale. Therefore, we were able to score positive fluorophore colocalization, if the fluorescence centres were within three pixels of each other, since this is well within the PSF. To prevent over-representation of non-damage-binding molecules in the analysis, when a tightrope was observed with a damage colocalized Qdot all remaining molecules on the same tightrope were excluded from analysis. Images were acquired at 10 fps for 5 s and stacked to provide a steady-state snapshot of colocalization, but without dynamic information. For each flow cell used, the system was calibrated to ensure correct colour alignment; further details are provided in the Supplementary Data.

#### Live cell imaging

The intracellular dynamics of protein motion provides an excellent indicator for whether the proteins are freely diffusing through solution or interacting with DNA. Diffusing molecules blend into the background whereas genome-associated molecules appear as fluorescence spots (36,37). Using this approach, fluorescent molecules were examined on a cell-by-cell basis and were categorized as not binding the genome if a homogenous distribution of fluorescence was observed. By contrast, the appearance of spots that persist for the duration of our movies (10 s) in a cell indicated that the Uvr-eGFP proteins were binding to the genome. Although the number of spots per cell varied, we classified a cell with one or more spots as static.

#### Statistics

‘*n*’ refers to the number of flow chambers used per experiment. Significance was determined using the Student’s *t-*test and consequent *P-*values are reported. Any error information not included in the results section can be found in Table 1 or the corresponding figure legend as indicated.

**Table 1.** NER complexes detected using a triple colour single-molecule tightrope assay

Uvr protein complex	Number Observed	Occurrence
A	230	61.5% (±3% SEM; *n* = 3)
B*	40	10.7% (±2% SEM; *n* = 3)
C	59	15.8% (±3% SEM; *n* = 3)
AB	25	6.7% (±0.6% SEM; *n* = 3)
AC**	8	2.1% (±1% SEM; *n* = 3)
BC	6	1.6% (±0.3% SEM; *n* = 3)
ABC***	6	1.6% (±0.6% SEM; *n* = 3)

*UvrB does not bind to DNA alone.

**UvrAC complexes were only observed with UvrB present in the flow cell.

***Triple-coloured complexes.

## RESULTS

### Investigating the heterogeneity of complex formation

**Figure 1.**
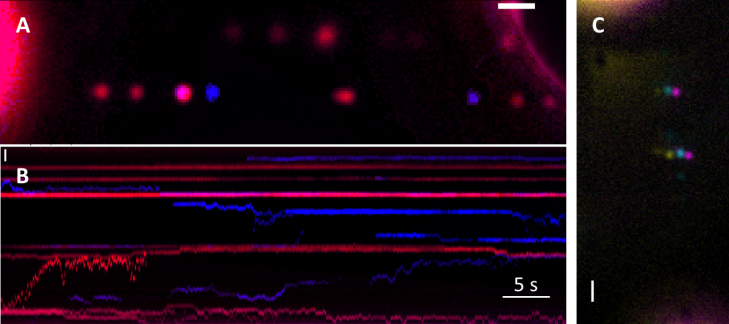
UvrABC as a repairosome complex. (**A**) Dual colour visualization of UvrABC complex, UvrAB (red) UvrC (blue) (**B**) Kymograph showing position versus time for the Qdots in (A), indicating complexes forming, dissociating and diffusing together. Dual coloured complexes suggest the formation of a repairosome (a breakdown of complexes formed can be found in Table 2). The heterogeneity of the complexes formed is clear. See Supplementary Movie S1. (**C**) A triple-coloured example of two moving repairosomes. The colours are horizontally offset to improve the clarity of the colocalization. In this image the DNA runs from top to bottom. See Supplementary Movie S2. Scale bars represent 1 μm.

To study the distribution of complexes formed by UvrA, UvrB and UvrC, we differentially labelled UvrA, UvrB and UvrC (1:1:0.5 nM respectively) with three different coloured Qdots (565, 605 and 655 nm), and incubated them together before adding to undamaged λ-DNA tightropes. All three colours were simultaneously imaged bound to DNA tightropes *in vitro* (Figure 1A), using a TripleSplit (Cairn Research) optimized to ensure no spectral bleed-through (see Supplementary Data). Because the DNA tightropes are lifted from the surface, we can be certain that any protein complexes formed are not due to the incidental overlap of proteins/Qdots that have non-specifically attached to the surface. As a result, it is not necessary to fluorescently tag the DNA, removing another potential complication. Of 374 Qdots examined the majority were UvrA alone (61.5%), followed by UvrC (15.8%) and UvrB (10.7%) (see Table 1 for a full summary). Consistent with previous studies (11,19,38), controls performed here indicated that UvrB alone was incapable of binding to DNA tightropes (data not shown). Therefore, the low percentage of UvrB molecules bound alone to DNA have non-fluorescent partners of either UvrA, UvrC or have bound and subsequently ejected UvrA (6,39). Complexes were also directly seen; UvrAB (6.7%) was most common and most rare were UvrAC (2.1%), UvrBC (1.6%) and UvrABC (1.6%). Motile examples of all complexes could be found, increasing confidence that the colocalization reflects real complex formation rather than fluorescence integral overlap due closely bound but non-coincident proteins.

For UvrABC, we found two out of the six triple-coloured complexes were moving (Figure 1C and Supplementary Movie S2). Because all three colours moved together this provided high confidence that the UvrABC complex was indeed being formed. It is important to note that UvrAC complexes were also observed. However, these complexes were only observed in the presence of UvrB indicating that the UvrAC complexes require non-fluorescent UvrB as a scaffold. The very low occurrence of UvrABC complexes is in part due to the probability of observing non-fluorescent Qdots (40), however there is also the possibility that the Qdot labelling reduces the affinity of the complex components for one another through steric hindrance. To reduce this potential of any labelling artifact, we first created UvrAB complexes on DNA tightropes with non-biotinylated UvrA and antibody Qdot-labelled UvrB. We then introduced differentially coloured streptavidin-Qdot-labelled biotin-UvrC and looked only for moving dual-coloured complexes to ensure colocalization was not artifactual. Figure 1B shows an example kymograph of Qdot_655_-labelled UvrB (red) complexed with unlabelled UvrA on a DNA tightrope. When UvrC (blue) was added to the flow cell, we were able to detect motile, static and UvrC complexed with UvrAB (see Table 2). Of 420 complexes examined, we found 67 were UvrABC where the blue and red fluorescence spatially overlapped, and of these 23% (±9% SEM; *n* = 6) moved together. Therefore, this two-coloured labelling methodology increased the total number of UvrABC complexes observed. To ensure that UvrA was still present in the complexes and to ensure that it was not the specific label that reduced colocalization, we reversed the labelling, so that UvrA was labelled in the initial UvrAB complex. The percentage of UvrABC movers showed no change (22 ± 2% SEM; *n* = 3). Altogether, these results indicate the previously postulated UvrABC repairosome (25,26) can form on undamaged DNA and linearly diffuse.

**Table 2.** Formation of UvrABC complex revealed using two different colour Qdots in the single-molecule tightrope assay

Uvr protein complex*	Number Observed	Occurrence
**AB**	371	48.4% (±4.6% SEM; *n* = 9***)
**C**	268	35.0% (±4.8% SEM; *n* = 9***)
ABC**	127	16.6% (±3.0% SEM; *n* = 9***)

UvrAB was labelled with a Qdot on either UvrA or UvrB (no difference to statistics was seen) and incubated with differentially labelled UvrC. UvrABC was counted as the formation of a dual colour complex.

*These data were collected from separate experiments with either UvrA or UvrB labelled. No significant difference in occurrence was observed (data not shown) indicating that when dual colour complexes were detected UvrABC was present.

**Of these dual colour complexes 24 were motile.

***Here, *n* refers to number of flow cells and was the value used to calculate SEM.

Another very important observation made from such multiple labelling experiments is the diversity of complexes formed. This is exemplified by the kymograph shown in Figure 1B and the corresponding movie (Supplementary Movie S1), where UvrC is labelled blue and UvrAB red (UvrA labelled). Here, it can be seen that UvrAB complexes can collide and diffuse together, and also bind to UvrC. These data indicate that complexes can be formed from numerous components and in various ways, either through diffusion along the DNA or directly from solution.

### Damage binding preference of specific protein complexes

**Figure 2.**
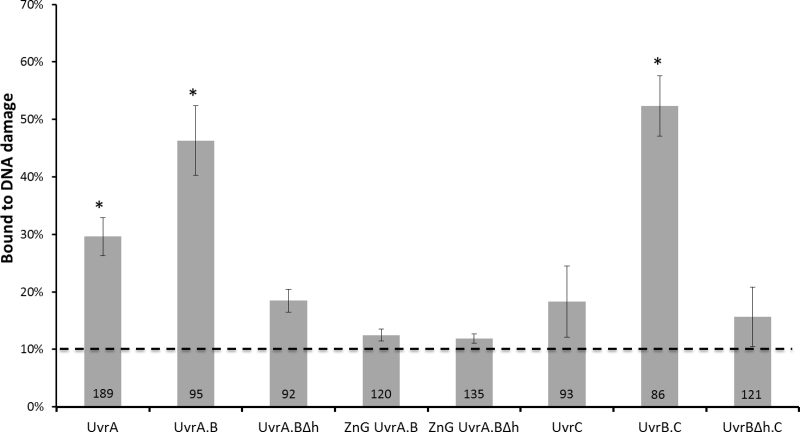
Probability of finding a Uvr protein complex colocalized with a damage marker. Values for mean probability percentage (±SEM, where, *n* refers to repeated experiments) bound to damaged DNA were 30% (±3% *n* = 5), 46% (±6% *n* = 4), 18% (±2% *n* = 4), 12.5% (±1% *n* = 3), 12% (±0.8% *n* = 3), 18% (±6% *n* = 3), 52% (±5% *n* = 4) and 15% (±5% *n* = 5) for UvrA, UvrA.B, UvrA.BΔh, ZnG UvrA.B, ZnG UvrA.BΔh, UvrC, UvrB.C and UvrBΔh.C, respectively. These data are summarized in Supplementary Table S1. The dashed line represents the probability (10.1%) of random association to damage based upon UvrA-Qdot binding to the mid-point of a DNA tightrope. *Indicates statistically significantly difference (*P* < 0.05) relative to the 10.1% random association value. UvrA.B and UvrB.C are not statistically different (*P* > 0.47). UvrA.BΔh, ZnG UvrA.B, ZnG UvrA.BΔh, UvrC and UvrBΔh.C are not statistically different to each other and the random association probability (*P* > 0.37). UvrA is statistically distinct from the UvrA.B/UvrB.C group (*P* < 0.01) and the Uvr mutant group with UvrC (*P* < 0.01).

Figure 1 demonstrates the diverse composition of a mixture of labelled complexes; however their precise role in NER is uncertain, therefore we set out to discover which of these were capable of finding a damaged nucleotide. Fluorescein-dT is known to elicit robust NER repair (41), therefore we incorporated a single such lesion into our 48.5 kbp λ-DNA tightropes and scored the probability of finding these complexes on damage. To indicate the location of damage, we placed a biotin locally and incubated with 10 nM streptavidin-coated Qdot565 for at least 10 min. These long-lived fluorophores were ideal for marking lesion position. Introduction of Qdot_655_-UvrA into the flow cell led to binding of UvrA across the length of the DNA tightropes including at damage markers. To prevent ‘over-labelling’ of the DNA such that the probability of colocalization with damage was over-represented, we only examined tightropes with six or fewer proteins bound, equivalent to a uniform spacing of >7.5 kbp or 2.5 μm, easily separable on our system (19). As shown in Figure 2, Qdot-UvrA molecules were found to bind to damage DNA with a frequency of 30% (±3% SEM; *n* = 5). By contrast, UvrAB complexes (singly labelled on UvrB with a Qdot_655_) were found to bind with a significantly (*P* < 0.05) greater probability to damage (46 ± 6% SEM; *n* = 4). This result agrees with previous observations that UvrAB complexes preferentially bind damage (39). In addition, the increased binding of UvrAB confirms that the approach taken to introduce damage is effective and the damage marking Qdot does not prevent the association of the complexes at the damage site, indeed it may offer a target for repair itself (42).

To understand the nature and specificity of the interaction with damage, we also studied a mutant UvrB with the tip of its ß-hairpin removed, which has been shown to be essential for identifying DNA damage (43) We examined the DNA damage binding capability of 92 UvrAB_Δβhairpin_ complexes and found only 18% (±2% SEM; *n* = 4) were colocalized with damage. This value is significantly lower (*P* < 0.05) than wild-type (WT) UvrAB (46%), confirming that fluorophore colocalization reflects detection of DNA damage. As an additional control, we established the background level of colocalization of proteins, i.e. the false positive threshold. We simply scored the probability of Qdot-UvrA binding to the midpoint of undamaged double-stranded DNA tightropes using the identical constraints for colocalization applied in this study (see ‘Material and Methods’ section). Analysis of 196 tightropes revealed the threshold as 10.1% (±1.1% SEM; *n* = 2). We also checked that this was not protein specific by performing similar controls with UvrBC and UvrC. The probability of Qdot-UvrBC colocalizing with the tightrope centre was found to be 11.3% (±1.4% SEM) and for Qdot-UvrC 9.9% (±6.4% SEM) consistent with Qdot-UvrA. This provides a lower baseline, any colocalization probabilities not significantly above this value are considered non-specific.

#### UvrA zinc-finger deletion reduces DNA damage detection

The probability of UvrA colocalising with sites of DNA damage is lower than UvrAB. However, because UvrA associates with damage significantly (*P* < 0.05) better than UvrAB_Δhairpin_ or the background level of colocalization, this suggests that UvrA may have intermediate damage specificity. To confirm this idea, we studied a mutant of UvrA in which the tip of the C-terminal zinc finger is removed (ZnG–UvrA). This C-terminal zinc-finger domain of UvrA plays a key role in damage detection and critically stimulates UvrB’s ATPase activity along with DNA damage (44–46). Of 120 ZnG–UvrAB complexes studied, only 12% (±1% SEM; *n* = 3) colocalized with damage. This value is significantly lower than UvrAB (*P* < 0.01), confirming that UvrA does bind damage in this assay, although with weaker affinity than in the presence of UvrB. Unsurprisingly, only 12% (±1% SEM; *n* = 3) of the double mutant complex of ZnG–UvrA and UvrB_Δhairpin_ were bound to damaged DNA.

#### UvrBC specifically binds to DNA damage

We previously showed the UvrBC complex can bind to dsDNA (19) and that alterations to the DNA-binding interface, either Tyr96Ala mutation or deletion of the β-hairpin of UvrB, affect motility of the whole complex. This suggests that UvrB is actively engaged with the DNA as part of the UvrBC complex. However, the potential role of this complex in NER is not understood. UvrBC has only been noted to bind specific damage containing substrates where the DNA structure is unpaired around the lesion (23,24). Again, to determine if UvrBC plays an active role in repair, we studied the interaction of both UvrC alone and in complex with UvrB. Of 93 observed UvrC DNA interactions, 18% (±6% SEM; *n* = 4) were found to colocalize with damaged DNA. This is not statistically different from baseline colocalization (10.1%, see above) suggesting no damage binding preference for UvrC. In stark contrast, 52% (±5% SEM; *n* = 4) of the 86 UvrBC interactions examined were found to colocalize with sites of damage. This surprising result suggests that UvrBC is capable of locating damage when embedded in long stretches of DNA. To provide secondary confirmation of this result, we were able to show that the UvrB_Δβhairpin_ mutant in complex with UvrC was incapable of detecting damage. Of 121 UvrB_Δβhairpin_C complexes studied, only 15% (±5% SEM; *n* = 5) colocalized with damage. This value is significantly different (*P* < 0.01) to WT UvrBC suggesting that the UvrBC complex interacts with damaged DNA and uses the β-hairpin of UvrB to distinguish damaged nucleotides. Remarkably, the damage colocalization probability for UvrAB and UvrBC complexes were not found to be statistically different (*P* = 0.4), further suggesting a potential role for the UvrBC complex in damage location.

Due to the complexity of making damaged tightropes on λ-DNA, it is not possible to be certain that the damaged oligonucleotide was fully ligated. However, to support that the lesions were ligated or that ligation is not important, we created an alternative tightrope with multiple damage locations by tandem ligating a damage containing oligonucleotide (see Supplementary Data). This approach results in >95% ligation efficiency (47,48) and using this damage substrate we found no difference in the ability of the NER proteins to locate damage. Furthermore, to rule out that UvrBC and UvrC were not simply binding to a single-stranded and double-stranded junction, a DNA construct was created that contained a single-stranded non-annealed region (see Supplementary Data) sandwiched between two *λ*-DNA molecules. UvrBC did not bind to this region with high probability 6% (±3.5% SEM; *n* = 4); similarly for UvrC only 3% (±3.2% SEM; *n* = 4) were found to colocalize. Both of these control values are below the random colocalization threshold (Supplementary Figure S2) suggesting that UvrBC is indeed locating damage on the DNA tightropes in an active ß-hairpin-dependent manner.

### Fluorescence imaging of UvrB or UvrC in *E. coli*

#### UvrA is not necessary for DNA damage binding by UvrB and UvrC

Our observation that a UvrBC complex can locate damage on DNA tightropes may suggest a role in repair *in vivo*. Therefore, we sought to determine if UvrB and UvrC respond to the presence of damage *in vivo* in the absence of UvrA. Fluorescently tagged proteins freely diffusing *in vivo* blend into the background leading to a homogenous spread of fluorescence, whereas previous studies have shown that when proteins bind to the genome *in vivo* their diffusion is slowed (33,36,49–51); resulting in fluorescent spots. Following this approach, we studied eGFP tagged UvrB and UvrC *in vivo* by ectopically expressing these protein fusions in a *uvrA* deleted background (UvrA^−^).

**Figure 3.**
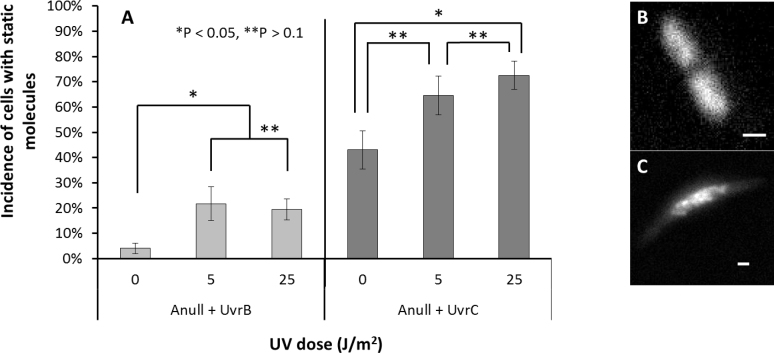
Genomic association of UvrB or UvrC is revealed by live cell fluorescence imaging. (**A**) The percentage of UvrA null cells complemented with UvrB with a static population of molecules were 4% (±2.1% *n* = 4), 22% (±6.7% *n* = 4), 19% (±4.1% *n* = 4), at 0, 5 and 25 J/m^2^ of UV (254 nm) exposure, respectively. For UvrA-null cells complemented with UvrC these values were 43% (±6.8% *n* = 3), 65% (±7.7% *n* = 9), 73% (±5.6% *n* = 8), at 0, 5 and 25 J/m^2^ UV (254 nm), respectively. Statistics reported are mean ± SEM, where, *n* refers to repeated experiments. (**B**) An example image of cells with homogenously diffusing proteins, (**C**) or static molecules indicating proteins bound to damaged DNA. Scale bars represent 1 μm.

We first performed controls to ensure that cells containing the ectopically expressed proteins were viable. We complemented UvrA^−^, UvrB^−^ and UvrC^−^ cells with their eGFP-tagged counterparts and exposed them to various UV doses. Cell growth for all null cells was severely attenuated following exposure to >5 J/m^2^ 254 nm UV radiation, whereas upon complementation, all cell types with their respective uvr-eGFP gene constructs were rendered viable up to 25 J/m^2^ (Supplementary Figure S3). Next, we determined the proportion of cells that possessed static eGFP-labelled proteins; indicative of DNA binding. In the absence of UV-induced damage, only 4% (±2.1% SEM; *n* = 4) of 100 UvrB-eGFP containing UvrA^−^ cells were static. The remaining cells appeared with a homogeneous background of fluorescence, consistent with proteins diffusing in the cellular cytoplasm. Mild UV exposure (5 J/m^2^) increased the static population to 22% (±6.7% SEM; *n* = 4) of 121 cells observed, significantly higher (*P* > 0.05) than unexposed cells. Further increasing the UV exposure to 25 J/m^2^ showed no increase in the static population (19% ± 4.1% SEM; *n* = 4) of 124 observed cells, suggesting the damage response is saturated at low levels of exposure. UvrC-eGFP behaved quite differently from UvrB-eGFP in UvrA^−^ cells, without UV 43% (±6.8% SEM; *n* = 3) of 86 cells possessed static UvrC-eGFP. As with UvrB, upon exposure to 5 J/m^2^ UV the static population rose to 65% (±7.7% SEM; *n* = 9) of 65 cells. However, unlike UvrB complementation, further exposure to UV damage (25 J/m^2^) resulted in an even higher damage response 73% (±5.6% SEM; *n* = 8) of 102 cells observed, statistically greater than the unexposed cells (*P* < 0.05). These results (Figure 3) indicate that UvrB and UvrC respond to DNA damage independently of UvrA *in vivo* and further strengthen our *in vitro* observations made with purified proteins.

### UV survival of UvrA-null cells complemented with Uvr proteins

#### UvrC overexpression confers improved survival in UvrA knockout cells exposed to UV

The observations made thus far suggest that not only can UvrBC complexes find damage, but in the absence of UvrA they are capable of associating with DNA in response to DNA damage. However, it is not clear that this UvrA-independent DNA damage response pathway is capable of facilitating damage processing. To test if this UvrA independent pathway is capable of repair, we studied the survival characteristics of cells exposed to UV.

**Figure 4.**
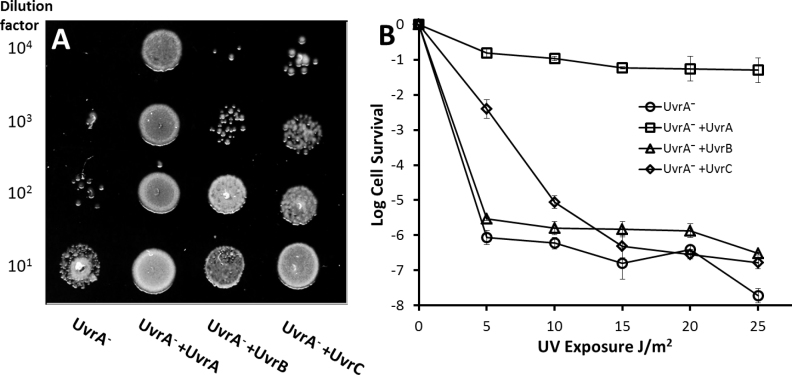
Survival of UvrA^−^ cells exposed to UV. (**A**) Spot plates of decreasing cell titers exposed to 5 J/m^2^ UV (254 nm) show improved survival with ectopic UvrB or UvrC. Lane 1 UvrA^−^ cells, lane 2 UvrA^−^ cells complemented with UvrA-eGFP, lane 3 UvrA^−^ cells complemented with UvrB-eGFP, lane 4 UvrA^−^ cells complemented with UvrC-eGFP. (**B**) Quantification of spot plates by colony counting. Survival of UvrA^−^ cells complemented with eGFP-tagged NER proteins versus UV dose shows a significant improvement in survival at low UV doses (5–10 J/m^2^) for UvrC-complemented cells. Cell survival is shown in logarithm units and error bars indicate standard deviation.

Our controls in this experiment were UvrA^−^ complemented with UvrA. Exposure of UvrA^−^ cells to 5 J/m^2^ UV light (254 nm) greatly impaired survival; however, ectopic expression of UvrA restored viability (Figure 4A, columns 1 and 2). Next, we complemented UvrA^−^ with UvrB-eGFP or UvrC-eGFP in which cells expressed 1373 and 183 molecules per cell, respectively (Supplementary Figures S4 and 5), and found that in both cases there was improved survival (Figure 4A, columns 3 and 4). For quantification of this effect, we generated survival curves by irradiating a number of cell dilutions with varying doses of UV and counting the colonies that grew following plating (Figure 4B). These values are presented as logarithmic growth relative to cells not exposed to UV, therefore as the magnitude of the negative value increases this indicates more compromised growth. As expected, UvrA complementation of UvrA^−^ showed the greatest level of UV resistance with −1.3 (equivalent to 5%) survival even after 25 J/m^2^ UV exposure. At 5 J/m^2^ UvrA^−^ cells with UvrB showed small, but significantly higher (*P* < 0.05) log relative cell survival of −5.5 versus non-complemented UvrA^−^ cells (−6.0; Figure 4B). The improved survival with ectopic UvrB was only observed at low doses of UV, at 10 J/m^2^ UvrA^−^ cells complemented with UvrB showed no significantly improved survival. In contrast to UvrB, UvrA^−^ cells ectopically expressing UvrC showed much greater survival than both UvrA^−^ and UvrA^−^+UvrB at low and moderate doses of UV. At 5 and 10 J/m^2^ UV exposure log relative survival of UvrA^−^+UvrC was recorded as −2.4 and −7 respectively; significantly better (*P* < 0.05) than the UvrA^−^ cells which showed −6.0 and −6.2 log relative survival at these UV exposures. At UV doses above 15 J/m^2^, the UvrC complemented cells showed no significant difference in survival from UvrB-complemented cells or UvrA-null cells indicating that UvrA is essential for survival even with additional UvrC present. The improved survivability conferred by UvrC is only significant at low to moderate UV doses.

## DISCUSSION

Nucleotide excision DNA repair is a multi-enzyme process that initially requires damage recognition followed by verification, incision, removal of the damaged product and finally DNA resynthesis. Numerous complexes have been proposed to form during NER in prokaryotes; however, their dynamics and specific roles remain uncertain. Single-molecule and ensemble approaches have been used previously to reveal the existence of UvrAB (4,6,9,11) and UvrBC (19,20,22–24) complexes, which likely represent the most populated forms *in vivo*. The UvrAB complex is well established in damage search; however a role for the UvrBC complex in a process other than DNA incision is not clear. Here, using a single-molecule *in vitro* system we show that UvrBC complexes specifically bind defined DNA lesions. To test if this also occurs *in vivo*, we were able to show that UvrB and UvrC migrate to the genome following UV damage in UvrA-null cells. Finally, by demonstrating improved UV survival of *E. coli* lacking UvrA when ectopically expressing UvrC, we can conclude that the UvrBC complex assists in DNA damage processing independently of UvrA.

### UvrABC can form a single ‘repairosome’ complex

To ensure reliable damage detection, NER utilizes kinetic proofreading, which involves the sequential verification of damage by subsequent partners in the repair pathway (18). A key aspect of proofreading is competition between the rate of complex release and the binding of the next component; however, the solution concentration of the next component in the pathway can vary. Formation of a super-complex or ‘repairosome’ including UvrA, UvrB and UvrC, as previously suggested (25,26), would greatly expedite partner recruitment. By labelling each component of the potential repairosome, we have been able to both directly image and statistically infer its existence. This approach has also enabled us to examine the dynamics of complex formation and decay. For example, we observed UvrC molecules joining pre-made UvrAB complexes to form the UvrABC repairosome (Figure 1). Having all of the NER components in a single complex clearly will improve the efficiency of NER. Although, with only 10–20 copies of UvrC present in the cell (52), this would limit the number of available repairosome complexes. However, efficient incision followed by UvrD-mediated recycling (15,16) will enable UvrC to become available for binding UvrAB complexes on DNA awaiting incision. Given UvrA has been shown to load multiple UvrB molecules (6), UvrC could also bind UvrB deposited at lesions (8). The insights gained here about how UvrC is capable of diffusing on and off a UvrAB complex suggests that UvrC’s 1D search mode (19) could facilitate rapid local repair of clustered damage events (53). The degree of Uvr complex heterogeneity observed in our visual tightrope assay is remarkable. Figure 1 shows multiple complexes forming, including UvrABC and dimeric UvrC. These complexes form and disintegrate on DNA; therefore a significant remaining challenge is to understand how each of these Uvr complexes contributes to repair both *in vitro* and *in vivo*.

### UvrAB is the primary damage detection complex

There is ample biochemical evidence to date that suggests UvrA and UvrB form a complex in solution, as either UvrA_2_UvrB or UvrA_2_UvrB_2_ (4–10). Given the excess of UvrB in cells (54), and given UvrB interacts more tightly than UvrA with DNA damage alone, UvrA is not expected to search alone for damage, unless in conditions where there are vast amounts of damage that overwhelm UvrB. At such times, UvrA can catalytically deposit UvrB on lesions as demonstrated *in vitro* (6), and recently confirmed using cellular imaging (51). Bulk biochemical analysis from several laboratories also suggest that UvrA does not bind damage with as high affinity as the UvrAB complex (39,55,56). Our single-molecule data confirm that UvrA is not as specific as the UvrAB complex (Figure 2), since it is seen to bind with lower probability to damage. The interaction of UvrA with damaged DNA is mediated in part by the C-terminal Zn finger containing region. Removal of this Zn finger region (ZnG–UvrA) attenuates UvrA’s ability to distinguish damage and load UvrB (44). We found that the ZnG–UvrA complexed with UvrB could not locate lesions, indicating that even its weak association to damage is crucial for lesion location. UvrA likely detects distortions in the double helix triggering UvrB to then examine the damage further (4,55); these events are coordinated by the multiple and distinct ATPase sites (51,57).

### UvrBC complexes locate DNA damage and may offer an alternative pathway for UV survival

UvrB and UvrC are known to form a complex to facilitate incision (8); however only recently has it been shown that a complex of UvrB and UvrC scans duplex DNA (19,20). Our *in vitro* DNA tightrope data indicate that this UvrBC complex is also capable of binding to lesions in the absence of UvrA. This unexpected observation was confirmed by removal of the damage sensing UvrB ß-hairpin, which ablated UvrB_Δhairpin_C’s capability of locating lesions (Figure 2). Previous bulk phase investigations indicated UvrB and UvrC form a complex in solution (23,24), via the UvrB C-terminal domain and homologous region in UvrC (22,58,59). However, this complex was shown to be incapable of incising damaged duplex DNA unless pre-processed with either a 3′ incision or a bubble around the damage (23,24). Therefore, we were surprised to see a modest increase of UvrB-eGFP binding to DNA in response to UV exposure in cells lacking UvrA. With 140–400 copies of endogenous UvrB per cell going up to ∼2000 following SOS (34,60) the levels far exceed those of endogenous UvrC (∼10), which is not SOS induced (52). Therefore if UvrB-eGFP is loaded by endogenous UvrC in response to UV, this may explain the relatively small but significant response (Figure 3). This also suggests that unlike UvrA, UvrC does not have the capacity to load multiple UvrB molecules at different damage sites (6).

We estimate that 180 molecules of UvrC-eGFP are expressed per cell from our ectopic constructs (see Supplementary Data). UV damage is estimated to generate ∼120 CPDs per *E. coli* genome at 5 J/m^2^ (2,61). Therefore, the additional 180 UvrC molecules would not be saturated at 5 J/m^2^, however at 10 J/m^2^ there would not be enough UvrC to cope with the damage. This is consistent the observed dramatic and UV dose-dependent DNA association of UvrC-eGFP *in vivo* (Figure 3), as UvrB binds UvrC and loads onto the genome.

In this study, we show loading of UvrBC directly onto damage without assistance from UvrA. Also, we demonstrate that UvrB and UvrC loading onto DNA *in vivo* has a marked effect on UV survival in the absence UvrA. This indicates a UvrBC complex forms *in vivo* and directly participates in DNA damage processing. How UvrBC is capable of participating in this new pathway is unclear. There is no evidence to date that suggests UvrBC alone is capable of excising damage. Alternatively, therefore, this complex will likely interact with the numerous UvrB-binding proteins *in vivo*, possibly photolyase, which is known to stimulate NER (62). Or, UvrBC complexes could help polymerases bypass sites of damage. Therefore, future studies are imperative to isolate the origin of this form of DNA processing. Nonetheless, the data presented here clearly reveal the existence of a novel processing pathway capable of dealing with low levels of damage. Given the normally enteric location of *E. coli*, exposure to UV is minimal and therefore this pathway may be used to deal with levels of damage that exceed the capacity of UvrA alone, prior to the SOS trigger.

## Supplementary Material

Supplementary Data
