# Supplementary material for: Recruitment of UvrBC complexes to UV-induced damage in the absence of UvrA increases cell survival
Source: Nucleic Acids Res. 2017 Dec 12;46(3):1256–65. doi: 10.1093/nar/gkx1244 (PMC5814901; doi:10.1093/nar/gkx1244)
Supplement: Supplementary Data [file gkx1244_supp.zip › nar-02453-d-2017-File007.docx]

**Supplementary Information for:**

**Recruitment of UvrBC complexes to UV-induced damage in the absence of UvrA increases cell survival**

Luke Springall^1,5^, Craig D. Hughes^2,5^, Michelle Simons^3,5^, Stavros Azinas^3^, Bennett Van Houten^4^ and Neil M. Kad^1,*^

**Proteins and buffers**

WT UvrA, UvrB and UvrC were purified as described previously (1-4). The zinc finger deletion (ZnG-UvrA) construct and the UvrB_Δβhairpin_ were also purified as described previously (2,4).

ABC buffer is comprised of 50 mM Tris-HCl (pH 7.5), 50 mM KCl, 1 mM ATP and 10 mM MgCl_2_ 10mM DTT.

**Constructing labeled, damaged DNA tightropes**

Lambda DNA is digested by Nt.BstNBI to create numerous nicks, only one pair of which is close enough together to generate an oligonucleotide fragment capable of spontaneous release (region 33776 – 33807 on lambda). Nicking was performed for 2 hours before a ten-fold excess of the replacement oligonucleotide (PHO-TTCAGAGZCTGAC-BIOTeg (where Z is fluorescein-dT and PHO is phosphorylated)) was added. Subsequently, the inserted phosphorylated oligonucleotide was ligated using T4 DNA ligase (New England Biolabs) overnight at room temperature with 1mM ATP. T4 DNA ligase was removed from DNA tightropes by washing with 25 flowcell volumes of 1M NaCl.

The long linear structure of lambda DNA makes determining the ligation state of damaged oligonucleotide uncertain. Therefore to ascertain the effect of ligation on the data presented in Figure 2 we utilized the fully ligated tandem damage substrate employed previously (5,6). A custom oligonucleotide with fluorescein and bio-dT (5’PHO-CCG AGT CAT TCC TGC AGC G(FldT)G TCC ATG GGA GTC AAA (BiodT)3’) was annealed into nicked pSCW01 plasmid (Geng et al., 2011) and then ligated with T4 DNA Ligase. Following this, the plasmids were linearized with XhoI and tandem ligated to make long DNA substrates for tightropes using fast-T4 DNA ligase. These produce regular damage sites at a distance of ~ 2 kb apart with >95% efficiency (Ghodke et al., 2014). Using this substrate we reproduced a subset of the data from Figure 2 as shown in Figure S1. As can be seen UvrAB and UvrBC are both statistically significantly higher in damage binding probability than UvrC, and the values compare well with Figure 2 of the main manuscript.

The addition of streptavidin coated Qdots (10 nM for 20 minutes) to the tightropes permitted visual localization of the damaged region. To confirm all biotins are labeled on DNA tightropes with Qdots we performed a control experiment to determine the colocalization of UvrA-eGFP; which does not possess a moiety with which to attach to the biotin on the DNA. Of 125 molecules studied 43 were colocalised with damage (34.4% ± 1.5%); this value is statistically identical (P = 0.26) to UvrA labelled with a Qdot where 29.6% ± 3.3% colocalised with damage. This indicates that either no biotins are free or any free biotins do not affect the colocalization statistics.

**Constructing DNA tightropes with a single stranded patch**

Single stranded patches were created through the ligation of the DNA oligonucleotide (5’GGGCGGCGACCT**GCGTGATCTTTGCCTTGCGACAGACTTCCTTGGCT**GGGCGGGCTGGC3’) to one cos end of lambda DNA. To the other cos end we ligated the shorter oligonucleotide (5’AGGTCGCCGCCCGCCAGCCCGCCC(TEG-bio)3’). Upon tandem ligation of these constructs a 35 base single stranded region (marked in bold) was created. The addition of streptavidin coated Qdots (10 nM for 20 minutes) to the tightropes permitted visual localization of the single stranded region. A tenfold excess of each oligonucleotide to lambda DNA was heated separately to 62 ⁰C for five minutes. The reaction was ligated overnight at room temperature and then for 24 hours 4 ⁰C with T4 DNA ligase. The ligation reactions were mixed and heated to 62 ⁰C for five minutes and allowed to ligate further overnight at room temperature with T4 DNA ligase. T4 DNA ligase was removed from DNA tightropes by washing with 25 flowcell volumes of 1M NaCl.

**Diagram showing the use of oligonucleotide bridges to create a single stranded region**

5’....GGGCGGCGACCT**GCGTGATCTTTGCCTTGCGACAGACTTCCTTGGCT**GGGCGGGCTGGC*gggcggcgacct*....3’

3’....*cccgccgctgga* CCCGCCCGACCGCCCGCCGCTGGA....5’

The long and short oligonucleotides are shown in capitals, the host lambda DNA in italics and the 35 base ss region in bold. The oligonucleotides were phosphorylated to ensure ligation and the short oligonucleotide possessed a 3’ biotin for Qdot conjugation.

**Fluorescence imaging**

We used the same protein labeling strategies as described previously (1,3,7); a 4:1 excess of Qdot to protein was used to ensure Qdots were singly labeled (1). Biotinylated avi-tagged Uvr proteins (UvrA and UvrC) were incubated separately with 565 nm, 605 nm or 655 nm streptavidin-conjugated quantum dots (Qdots; Invitrogen) in ABC buffer for 30 minutes prior to dilution to ~1 nM immediately prior to imaging. 1 µM N-terminal hemagglutinin tagged (HA) UvrB was added to 1 µM HA antibody and conjugated to 3 µM of Qdots using an antibody sandwich as described in (1) and diluted to ~1 nM immediately prior to imaging. UvrAB, UvrA_ZnG_B (2), UvrA_ZnG_B_Δβhairpin_, UvrBC and UvrB_Δβhairpin_C (4) complexes were labeled with Qdots via the UvrB only. This ensured that only proteins complexed with UvrB were visualized since UvrB is unable to bind to DNA alone (3,7,8). We did not always label the same protein with the same color tag to ensure that this did not introduce any bias.

To observe protein-DNA interactions, undamaged and damaged DNA substrates were used (5,9,10). We imaged DNA tightropes in a custom built flow cell with an mPEG_5000_ N-succinimidyl propionate (Sigma-Aldrich) blocked surface as described previously (7). Briefly, 5 µm diameter poly-L-lysine (Sigma-Aldrich) coated silica spheres (Polysciences Inc.) were flushed into the flowcell and allowed to adhere to the surface. 5 ng/µl DNA was subsequently passed through the flowcell at 300 µl/min which led to individual strands being suspended between the coated silica spheres, thus forming DNA tightropes (11). After washing through with ABC buffer, Qdot-tagged proteins were introduced and visualized.

The same optical platform was used for imaging single molecules on DNA tightropes and single molecules in cells. We performed oblique angle fluorescence (OAF) microscopy using our custom built microscope (7,11). This system is built on an Olympus IX50 frame with a custom excitation path consisting of a 488 nm JDSU DPSS laser source beam expanded 17.5x. This beam is focused using a 250 mm plano-concave lens to the back focal plane of an Olympus 1.45NA 100x objective lens. This generates a collimated beam at the sample plane and the lateral position of the focal point at the back aperture defines the exit angle of the excitation beam; this was guided to just below the critical angle. Because the Qdots used in this study can all be excited by this laser a long pass 500 nm dichroic was the only filter needed in the nosepiece; this is also ideal for imaging eGFP. To obtain triple color images we used an Optosplit III (Cairn Research, UK) optimized for 565 nm, 605 nm or 655 nm Qdots (see below) to separate the image into three channels which were projected and recorded using a DU897 EMCCD camera (Andor, Belfast, UK) at 10 fps for up to 60s. Live cell imaging was performed using the Hamamatsu Orca Flash4.0 V2 at the same frame rate on the same system for a maximum period of 20s with the exception of photobleaching acquisitions which continued until all fluorescence reached background levels. The camera pixel sizes were equivalent to 75 nm (Andor) and 63.2 nm (Hamamatsu) respectively in the sample plane corresponding to a magnification of 213x and 103x respectively (the difference is due to a change in the relay optics contained within the Optosplit III).

**Quantum dot colocalization**

To study the colocalization of damage or complex formation it was necessary to accurately align the color channels from the three-color image splitter (TripleSplit, Cairn Research). We divided the camera output into three sections each corresponding to the same field of view but separated by color using Chroma 630DCLP and T585LPXP dichroic mirrors. In addition, to eliminate spectral bleed-through we also used D655/40m and D565/40m band-pass filters. Fiducial markers on the coverslip surface were located that clearly emit across all colors. Such alignment markers included the surface silica beads that accumulate Qdots during the course of an acquisition; in addition, non-specific markers on the surface (most likely dust particles) were found that could emit across all channels. For every flowcell an alignment was performed for every acquisition in post-process using the fiducial markers and a custom written ImageJ macro.

To determine the precision of colocalization we randomly selected 20 images that we classified as colocalized and used super-localization (precision = 8.7 nm (~26 bp) (3)) to determine the positional offset between the colors. To do this we took the aligned images and drew a 49-pixel square box with the fluorophores approximately in the center. These images were color separated using ImageJ and then for each color we used the ImageJ GaussianFitOnSpot plugin to determine the center of the point spread function. By comparing fluorophores between all three channels used in this study we found a precision of 115 nm (± 14 nm SEM; n = 20).

**Ectopic protein expression levels**

In all live cell imaging and survival experiments Uvr proteins were transformed into the null cell line stated and glycerol stocks used as starters for subsequent investigations. Uvr Protein-eGFP expression was regulated by the T5-lac promoter on the pCA24N plasmid (12) with the lacI^q^ for strict suppression. In this study we did not induce protein expression. In order to quantify the amount of protein present we performed a direct measure of the number of UvrC molecules present in a population of cells and then performed absorption spectrometry to establish the relative protein levels for all other Uvr-eGFP proteins expressed.

*Direct assessment of the number of UvrC proteins*

After growing UvrA-null cells ectopically expressing UvrC-eGFP and plating onto agarose pads as described in the manuscript we visualized the photobleaching of individual cells. The cells were identified and focused using brightfield to minimize photobleaching and then recording was initiated prior to activation of the illumination laser at 488 nm. The total intensity of fluorescence for each cell followed a single exponential decay (Figure S4A). Towards the end of the decay transient there would be just a few molecules of UvrC-eGFP remaining; we used this region to quantify the stepwise photobleaching of individual fluorophores (Figure S4B). The data from the last step to background resulted in a biphasic distribution that was fitted to the sum of two Gaussians (Figure S4C) consisting of background noise and fluorescence from a single UvrC-eGFP. The mean shift of the signal relative to noise was plotted for 16 molecules as a second histogram and fitted to a single Gaussian distribution. The mean of this distribution provided the average fluorescence intensity of a single UvrC-eGFP (30 ± 2.7, SEM, n = 16). To calculate the number of molecules per cell, we fit the full photobleach decay to a single exponential and divided the amplitude by the average fluorescence intensity of a single UvrC-eGFP. A histogram of 42 measurements was fit to a single Gaussian with a mean of 183 ± 5 (SEM) molecules of UvrC-eGFP per cell expressed ectopically (Figure S4D).

*Spectrometric determination of the number of any Uvr proteins*

By growing cells to a specific density and then measuring their peak fluorescence intensity at 509 nm (Figure S5) we were able to determine the relative quantities of eGFP. Using UvrC-eGFP as a control the absorption of UvrA-eGFP was 4.5 fold higher, therefore equivalent to ~820 molecules and UvrB was 7.5 times higher equivalent to ~1400 molecules.

**References**

1. Wang, H., Tessmer, I., Croteau, D.L., Erie, D.A. and Van Houten, B. (2008) Functional characterization and atomic force microscopy of a DNA repair protein conjugated to a quantum dot. *Nano Lett*, **8**, 1631-1637.

2. Croteau, D.L., DellaVecchia, M.J., Wang, H., Bienstock, R.J., Melton, M.A. and Van Houten, B. (2006) The C-terminal zinc finger of UvrA does not bind DNA directly but regulates damage-specific DNA binding. *J Biol Chem*, **281**, 26370-26381.

3. Hughes, C.D., Wang, H., Ghodke, H., Simons, M., Towheed, A., Peng, Y., Van Houten, B. and Kad, N.M. (2013) Real-time single-molecule imaging reveals a direct interaction between UvrC and UvrB on DNA tightropes. *Nucleic Acids Res*, **41**, 4901-4912.

4. Skorvaga, M., Theis, K., Mandavilli, B.S., Kisker, C. and Van Houten, B. (2002) The beta -hairpin motif of UvrB is essential for DNA binding, damage processing, and UvrC-mediated incisions. *J Biol Chem*, **277**, 1553-1559.

5. Ghodke, H., Wang, H., Hsieh, C.L., Woldemeskel, S., Watkins, S.C., Rapic-Otrin, V. and Van Houten, B. (2014) Single-molecule analysis reveals human UV-damaged DNA-binding protein (UV-DDB) dimerizes on DNA via multiple kinetic intermediates. *Proc Natl Acad Sci U S A*, **111**, E1862-1871.

6. Kong, M., Liu, L., Chen, X., Driscoll, K.I., Mao, P., Bohm, S., Kad, N.M., Watkins, S.C., Bernstein, K.A., Wyrick, J.J. *et al.* (2016) Single-Molecule Imaging Reveals that Rad4 Employs a Dynamic DNA Damage Recognition Process. *Mol Cell*.

7. Kad, N.M., Wang, H., Kennedy, G.G., Warshaw, D.M. and Van Houten, B. (2010) Collaborative dynamic DNA scanning by nucleotide excision repair proteins investigated by single- molecule imaging of quantum-dot-labeled proteins. *Mol Cell*, **37**, 702-713.

8. Kacinski, B.M. and Rupp, W.D. (1981) E. coli uvrB protein binds to DNA in the presence of uvrA protein. *Nature*, **294**, 480-481.

9. Kochaniak, A.B., Habuchi, S., Loparo, J.J., Chang, D.J., Cimprich, K.A., Walter, J.C. and van Oijen, A.M. (2009) Proliferating cell nuclear antigen (PCNA) uses two distinct modes to move along the DNA. *J Biol Chem*.

10. Kuhn, H. and Frank-Kamenetskii, M.D. (2008) Labeling of unique sequences in double-stranded DNA at sites of vicinal nicks generated by nicking endonucleases. *Nucleic Acids Res*, **36**, e40.

11. Springall, L., Inchingolo, A.V. and Kad, N.M. (2016) DNA–Protein Interactions Studied Directly Using Single Molecule Fluorescence Imaging of Quantum Dot Tagged Proteins Moving on DNA Tightropes. *Chromosome Architecture: Methods and Protocols*, 141-150.

12. Kitagawa, M., Ara, T., Arifuzzaman, M., Ioka-Nakamichi, T., Inamoto, E., Toyonaga, H. and Mori, H. (2005) Complete set of ORF clones of Escherichia coli ASKA library (a complete set of E. coli K-12 ORF archive): unique resources for biological research. *DNA Res*, **12**, 291-299.

Figure S1

**Figure S1. Probability of finding a Uvr protein complex colocalised with a damage marker on tandem plasmid substrate.** Values for mean probability percentage (±SEM, where n refers to repeated experiments) bound to damaged DNA were 40% (± 8% n=5), 41% (± 2% n=4), 7% (± 0.5% n=2), for UvrA.B, UvrB.C and UvrC respectively. The numbers on the graph are the total number of molecules investigated for each condition. These values compare with figure 2 as follows: 46% (± 6%), 52% (± 5% n=4) and 18% (± 6% n=3) for UvrA.B, UvrB.C and UvrC respectively.

Figure S2


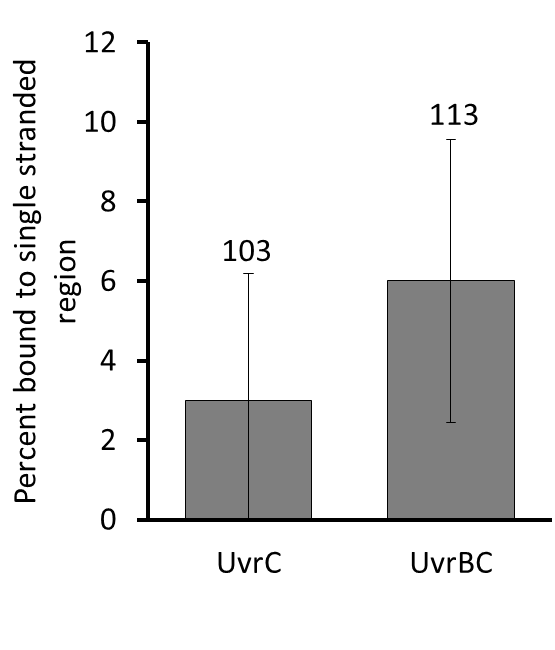


**Figure S2. Propensity of UvrC and UvrBC to bind single stranded regions.**

UvrC bound with 3% (± 3.2% n=3) probability to a single stranded region, and UvrBC bound with 5% (± 2.9% n=3) probability. These values represent mean ±SEM, where n refers to number of molecules examined as indicated above the bar. Both values are below the colocalisation threshold of 10.1% (see main text).

Figure S3


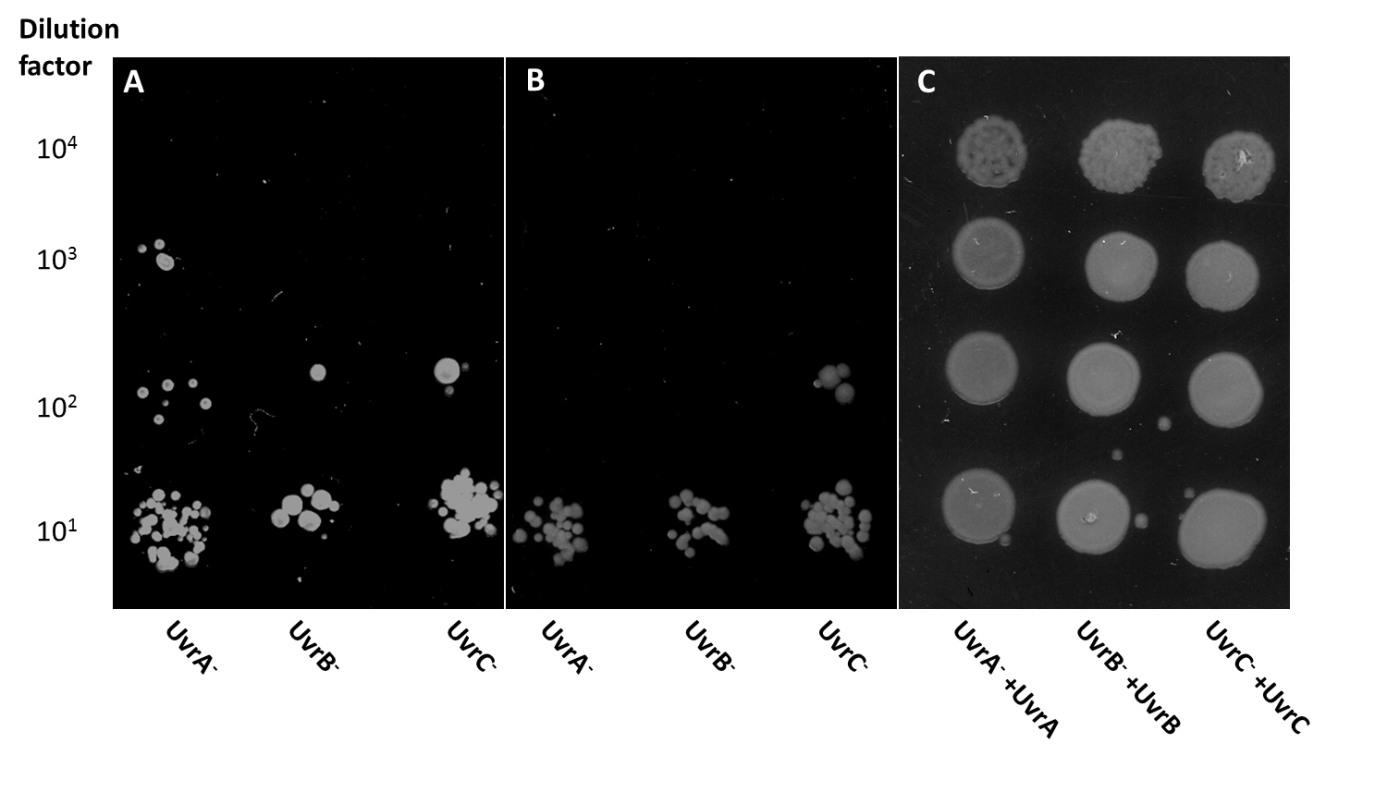


**Figure S3. Survival of UvrA^-^, UvrB^-^ and UvrC^-^ cells exposed to UV damage*.* (A)** Colony growth for null cells exposed to 5 J/m^2^, **(B)** or 10 J/m^2^. **(C)** Survival of null cells ectopically complemented with eGFP labelled Uvr proteins before exposure to 25 J/m^2^ 254 nm UV.

Figure S4


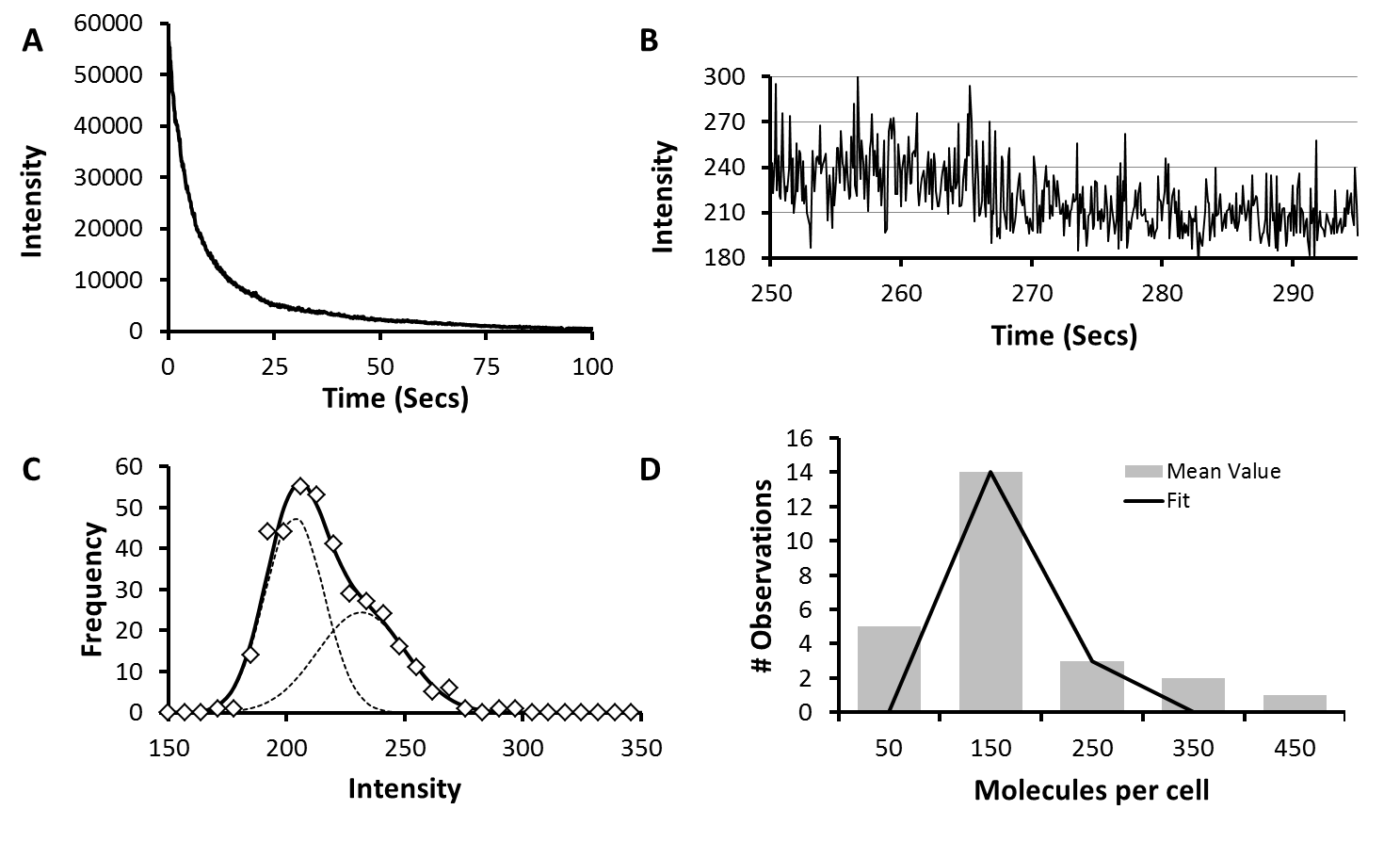


**Figure S4. Quantifying UvrC-eGFP *in vivo***. **(A)** Typical single exponential photobleaching decay curve for a single cell. **(B)** Close-up of the last step to background from a photobleaching decay curve. **(C)** Data from (B) re-plotted as a histogram, showing the two components, noise and last step fitted to two Gaussians (dashed lines). **(D)** Histogram of the number of molecules per cell fitted to a Gaussian distribution to yield an average of 183 ± 5.4 (SEM; n = 42).

Figure S5


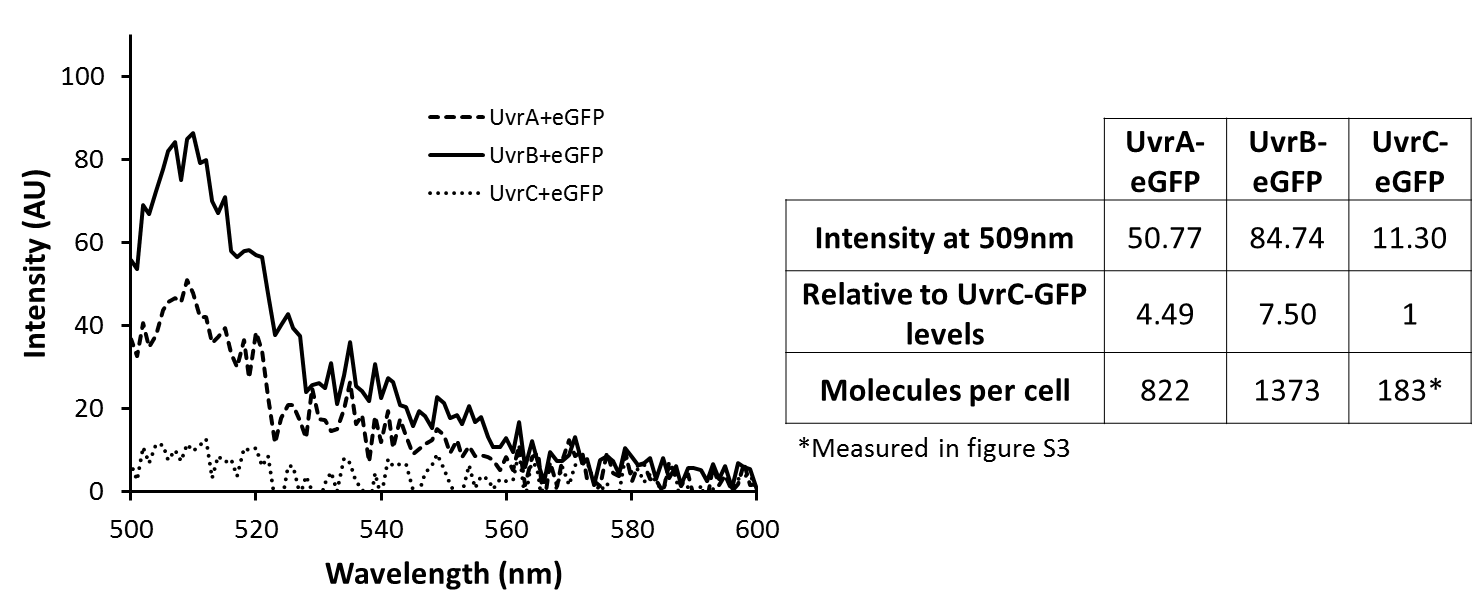


**Figure S5.** **Ectopic levels of C-terminally tagged eGFP proteins in UvrA^-^ cells.**

UvrA-null cells complemented with ectopically UvrA-eGFP, UvrB-eGFP or UvrA-eGFP were grown to OD_600_ 0.6 and lysed. Emission spectra are shown in the graph for each sample with excitation at 490 nm. The table shows the relative intensity of each eGFP labelled Uvr protein at the peak emission wavelength (509 nm). To remove the background auto-fluorescence, cells complemented with a non-GFP labelled UvrA were measured under identical conditions and the spectrum subtracted from the Uvr-eGFP tagged protein spectra.

**Table S1**

**Summary table for Statistics of Single Molecule damage Colocalization experiments**

|  | UvrA | UvrAB | UvrAB_Δh_ | UvrA_Zn_B | UvrC | UvrBC | UvrB_Δh_C | UvrA_Zn_B_Δh_ |
| --- | --- | --- | --- | --- | --- | --- | --- | --- |
| %Bound to damage | 29.63% | 46.32% | 18.48% | 12.50% | 18.28% | 52.33% | 15.70% | 11.85% |
| SEM* | 3.29% | 6.03% | 1.98% | 1.02% | 6.20% | 5.26% | 5.15% | 0.80% |
| n (Flow Cell) | 5 | 4 | 4 | 3 | 3 | 4 | 5 | 3 |
| Molecules observed | 189 | 95 | 92 | 120 | 93 | 86 | 121 | 135 |

* SEM values were calculated using the number of flow cells {n(Flow Cell)} not molecules observed

Δh refers to UvrB_Δβhairpin_, UvrB with the ß-hairpin removed

Zn refers to ZnG-UvrA, UvrA with the C-terminal zinc finger removed
